# Machine Learning Models for the Diagnosis and Prognosis Prediction of High-Grade B-Cell Lymphoma

**DOI:** 10.3389/fimmu.2022.919012

**Published:** 2022-05-24

**Authors:** Hui Kong, Haojie Zhu, Xiaoyun Zheng, Meichen Jiang, Lushan Chen, Lingqiong Lan, Jinhua Ren, Xiaofeng Luo, Jing Zheng, Zhihong Zheng, Zhizhe Chen, Jianda Hu, Ting Yang

**Affiliations:** ^1^ Department of Hematology, Fujian Institute of Hematology, Fujian Provincial Key Laboratory of Hematology, Fujian Medical University Union Hospital, Fuzhou, China; ^2^ Department of Pathology, Fujian Medical University Union Hospital, Fuzhou, China; ^3^ Department of Hematology, The Second Hospital of Longyan, Longyan, China

**Keywords:** high-grade B-cell lymphoma, clinical characteristics, diagnostic predictor, machine learning, classification models

## Abstract

High-grade B-cell lymphoma (HGBL) is a newly introduced category of rare and heterogeneous invasive B-cell lymphoma (BCL), which is diagnosed depending on fluorescence *in situ* hybridization (FISH), an expensive and laborious analysis. In order to identify HGBL with minimal workup and costs, a total of 187 newly diagnosed BCL patients were enrolled in a cohort study. As a result, the overall survival (OS) and progression-free survival (PFS) of the HGBL group were inferior to those of the non-HGBL group. HGBL (n = 35) was more likely to have a high-grade histomorphology appearance, extranodal involvement, bone marrow involvement, and whole-body maximum standardized uptake (SUVmax). The machine learning classification models indicated that histomorphology appearance, Ann Arbor stage, lactate dehydrogenase (LDH), and International Prognostic Index (IPI) risk group were independent risk factors for diagnosing HGBL. Patients in the high IPI risk group, who are CD10 positive, and who have extranodal involvement, high LDH, high white blood cell (WBC), bone marrow involvement, old age, advanced Ann Arbor stage, and high SUVmax had a higher risk of death within 1 year. In addition, these models prompt the clinical features with which the patients should be recommended to undergo a FISH test. Furthermore, this study supports that first-line treatment with R-CHOP has dismal efficacy in HGBL. A novel induction therapeutic regimen is still urgently needed to ameliorate the poor outcome of HGBL patients.

## Introduction

Diffuse large B-cell lymphoma (DLBCL) is an aggressive, highly heterogeneous type of lymphoma characterized by various clinical features and outcomes. Among DLBCL patients, some harbor not only morphological features of DLBCL but also MYC, Bcl-2, and/or Bcl-6 rearrangements. Based on the 2016 revision of the WHO classification of lymphoid neoplasms (1), these patients are classified as having high-grade B-cell lymphoma (HGBL), an extra-aggressive disease with complex karyotype and a series of pathomorphological features. Currently, HGBL is subgrouped as HGBL with MYC and Bcl-2 and/or Bcl-6 rearrangements, so-called double- or triple-hit lymphoma (HGBL-DH or HGBL-TH, respectively) (2) and as HGBL, not otherwise specified (HGBL-NOS), which lacks MYC and Bcl-2 and/or Bcl-6 rearrangements. HGBL is diagnosed using immunohistochemistry (IHC) and cytogenetic fluorescence *in situ* hybridization (FISH) of excised pathological tissues (3) such as the lymph node, bone marrow, and spleen (4). In addition, another entity, called “double-expressor lymphoma” (DEL) (5, 6), has been identified based on MYC and Bcl-2 protein overexpression by IHC without gene aberrations (MYC and Bcl-2 and/or Bcl-6 rearrangements) by FISH, with positive cut-off values for MYC+ of ≥40% and Bcl-2+ of ≥50% in most studies (7, 8). Although not as malignant as HGBL-DH, DEL is generally invasive, more common, and likely to have a better prognosis than HGBL-DH (5, 9, 10).

These new entities, defined by biological and histological peculiarities, have a well-known worse outcome, especially HGBL-DH/TH, and indicate a diagnostic and therapeutic difficulty for pathologists and clinicians.

Nevertheless, due to limited research, a uniform international consensus on which DLBCL should be detected with FISH, the only diagnostic method for HGBL-DH or HGBL-TH, has not yet been reached. Furthermore, predictive markers are lacking, and it is controversial to regard DEL as a predictor of HGBL-DH or HGBL-TH, although there is a certain degree of overlap between them (7, 8, 10). Considering the difficulties of routinely screening HGBL using FISH and the dismal prognosis of HGBL-DH or-TH, this retrospective study herein aims to compare and analyze the data from patients identified as HGBL and non-HGBL based on FISH analysis and to define valuable diagnostic predictors to build a diagnosis prediction model, setting the stage for further molecular genetic analysis of B-cell lymphoma patients with high-risk factors.

## Materials and Methods

### Patients

Data from 187 patients with aggressive mature B-cell lymphomas (including 152 cases with DLBCL and 35 cases with HGBL-DH/TH/NOS) followed up at the Fujian Medical University Union Hospital between April 1, 2018, and April 1, 2022, were retrospectively collected.

### Specimen Processing, Fluorescence *In Situ* Hybridization, and Immunohistochemistry

The pathological specimens from all cases were excised, stored as fresh-frozen or formalin-fixed paraffin-embedded (FFPE) tissues, and later analyzed by FISH and IHC. MYC, Bcl-2, or Bcl-6 translocation was detected by FISH using DNA probes annealing to specific sequences of the target genes and the ThermoBrite FISH slide processing system, strictly following the manufacturer’s instruction. The dual-color Break Apart rearrangement probes, namely, Vysis LSI MYC (8q24.21) (Cat# 05J91-001), Vysis LSI Bcl-2 (18q21.33) (Cat# 07J75-001), and Vysis LSI Bcl-6 (3q27.3) (Cat# 01N23-020) (ASR), and the ThermoBrite system were purchased from Abbott Laboratories (Chicago, IL, USA). For IHC staining, samples were probed with primary monoclonal antibodies against CD10 (clone SP67; Cat# 790-4506, Roche Tissue Diagnostics, Oro Valley, AZ, USA), MUM-1 (clone MRQ-43; Cat# 760-4529, Roche Tissue Diagnostics), Bcl-2 (clone SP66; Cat# 790-4604, Roche Tissue Diagnostics), c-MYC (clone Y69; Cat# 790-4628, Roche Tissue Diagnostics), Bcl-6 (RTU clone GI191E/A8; Cat# 760-4241, Roche Tissue Diagnostics), and Ki67 (clone 30-9; Cat# 790-4286, Roche Confirm); the primary antibodies were probed with the anti-rabbit or mouse secondary antibodies labeled with horseradish peroxidase (HRP). All staining was performed using the Ventana Benchmark ULTRA IHC staining module (Ventana, Tucson, AZ, USA). Each pathology report included H&E-stained sections and FISH, reviewed by the senior lymphoma pathologists in the Pathology Department based on the 2016 WHO lymphohematopoietic system tumor classification.

### Diagnosis, Staging, and Prognostic Index Score

All DLBCL cases with MYC and Bcl-2 and/or Bcl-6 rearrangements were diagnosed as HGBL-DH or HGBL-TH. Cases that appear blastoid or intermediate between DLBCL and Burkitt’s lymphoma (BL) that lacked MYC and Bcl-2 and/or Bcl-6 rearrangements were classified as HGBL-NOS. The cell of origin (COO) was defined according to the Hans algorithm (11), which is used to classify cases as germinal center B cell (GCB) or non-GCB depending on the expression of CD10, Bcl-6, and MUM-1 assessed by IHC. Patients were divided into DEL and non-DEL based on overexpression positivity cutoff for Bcl-2 or MYC of ≥50% or ≥40% of stained cells, respectively (6, 12). The Ann Arbor staging classification system (13), revised by Cotswold et al. in 1989 (14) (**Table S1**), for the risk group converted by the International Prognostic Index (IPI) (15) was used to evaluate the staging and prognosis of all patients (**Table S2**).

### Assessment of Clinical Features

Patient clinical characteristics included gender, age, white blood cell (WBC) count, serum lactate dehydrogenase (LDH) level, β2 microglobulin, Ann Arbor staging, A or B symptoms, IPI score, risk group, extranodal involvement sites (especially bone marrow), histomorphology, chromosome karyotype, immunophenotype (such as CD10, MYC, Bcl-2, Bcl-6, and MUM-1), DEL, Ki-67 proliferation index, baseline whole-body maximum standardized uptake (SUVmax), and the Epstein–Barr virus-encoded small nuclear RNA (EBER). Among the above characteristics, the Ann Arbor staging (13) and IPI score (15) were previously described. For cases in which the chromosome karyotype was available, a cytogenetic complexity score was calculated. Any numerical or structural abnormality, except for the translocations involving 3q27, 8q24, or 18q21, was counted as 1 event each. Cases with a cytogenetic complexity score >2 were considered to have a complex karyotype (16, 17). SUVmax before first induction was used to evaluate the functional metabolisms of tumors when diagnosing.

### Chemotherapeutic Regimens

The first-line chemotherapy treatment used was mainly the R-CHOP regimen (rituximab, cyclophosphamide, doxorubicin, vincristine, and prednisone). The second-line regimens included R-CHOP + X (X being lenalidomide, chidamide, zanubrutinib, or others), R-DA-EPOCH (rituximab, dose-adjusted etoposide, prednisone, vincristine, cyclophosphamide, and doxorubicin), R-DA-EDOCH (rituximab, dose-adjusted etoposide, dexamethasone, vincristine, cyclophosphamide, and doxorubicin), and R-HyperCVAD (rituximab, hyper-fractionated cyclophosphamide, vincristine, doxorubicin, and dexamethasone).

### Efficacy and Follow-Up

According to the Lugano Lymphoma Efficacy Criteria (18, 19), ^18^F-fluorodeoxyglucose (FDG) PET/CT or enhanced CT was used to evaluate disease status based on the Deauville score (20). Imaging evaluation was performed before the first induction chemotherapy and after every four courses of chemotherapy until the end of follow-up on April 1, 2022.

Efficacy was divided into complete remission (CR), partial remission (PR), stable disease (SD), and progressive disease (PD). The objective response rate (ORR) was calculated according to the percentage of CR+PR patients among all patients. Duration of remission (DOR) was defined as the period from the occurrence of the first CR to disease relapse or death due to any cause.

### Statistical Analysis

The data were analyzed with the SPSS V.26.0, Python V.3.9.0, and R 4.1.1 statistical software. A p < 0.05 was considered statistically significant. Student’s t-test was used for metric variables conforming to the normal distribution, while the Mann–Whitney U test was used for fitting non-normal distribution. The disorderly classification variables between two groups were analyzed by Pearson’s chi-square test (Fisher’s exact probability method was used when necessary). Survival rates including overall survival (OS) and progression-free survival (PFS) were estimated using the Kaplan–Meier method. The least absolute shrinkage and selection operator (LASSO) method (21) for high-dimensional data reduction is used to select the best predictive features of risk factors from HGBL patients (22). Machine learning algorithms, including Gradient Boosting Classifier, CatBoost Classifier, Random Forest Classifier, Extra Trees Classifier, Extreme Gradient Boosting, Logistic Regression, Decision Tree Classifier, Ridge Classifier, Ada Boost Classifier, K Neighbors Classifier, SVM-Linear Kernel, Naive Bayes, and Quadratic Discriminant Analysis, were used to establish prediction models. The 187 cases were split into a training set containing 70% of the observations and a test set containing the remaining 30%. For several models, the area under the curve (AUC), confusion matrix, precision, recall, and F1 value were used to evaluate the models (see details in the **Supplementary Material**).

## Result

### General Clinical Features

A total of 187 patients were included in this study, with 105 men (56.1%) and 82 women (43.9%) and an average age of 55.50 (± 15.02) years. All had high-risk factors, including but not limited to advanced Ann Arbor stage, extranodal involvement, double expressor, high intermediate, and high IPI risk group. Thirty-five cases out of 187 tumors had been classified as HGBL. Among them, HGBL-DH was the most common, with 3 cases of MYC/Bcl-2 HGBL-DH (8.6%, including 2 cases complicated with follicular lymphoma) and 21 cases of MYC/Bcl-6 HGBL-DH (60.0%, including 1 case complicated with the hemophagocytic syndrome). Four cases were HGBL-TH (11.4%), and 7 were HGBL-NOS (20%). Among the 187 patients, 135 were in the advanced Ann Arbor stage (29 of them were HGBL), 123 cases had extranodal involvement (28 cases were HGBL), 91 cases were with double expressor (20 cases were HGBL), and 112 cases were with high intermediate and high IPI risk group (26 cases were HGBL). Other essential features and IHC phenotypes are detailed in **Table 1**. Karyotypes were available for 18 out of 35 HGBL patients and 69 out of 152 non-HGBL patients. Among them, 2 cases in the former and 7 cases in the latter showed complex chromosomal aberrations (**Table S3**).

**Table 1:** Clinical characteristics of all patients.

Characteristics	HGBL (n = 35)			Non-HGBL (n = 152)	p-Value
	HGBL-DH (n = 24)	HGBL-TH (n = 4)	HGBL-NOS (n = 7)		
**Gender**					0.316
**Male**	13 (37.1%)	0 (0%)	4 (11.4%)	88 (57.9%)	
** Female**	11 (31.5%)	4 (11.4%)	3 (8.6%)	64 (42.1%)	
**Age**					0.182
** >60**	9 (25.7%)	0 (0%)	3 (8.6%)	71 (46.7%)	
** ≤60**	15 (42.9%)	4 (11.4%)	4 (11.4%)	81 (53.3%)	
**Ann Arbor stage**					0.118
** I–II**	1 (2.9%)	3 (8.6%)	2 (5.7%)	46 (30.3%)	
** III–IV**	23 (65.6%)	1 (2.9%)	5 (14.3%)	106 (69.7%)	
**B symptom**					0.436
** Yes**	7 (20.0%)	1 (2.9%)	3 (8.6%)	38 (25.0%)	
** No**	17 (48.5%)	3 (8.6%)	4 (11.4%)	114 (75.0%)	
**IPI score**					0.054
** <2**	2 (5.7%)	3 (8.6%)	4 (11.4%)	66 (43.4%)	
** ≥2**	22 (62.8%)	1 (2.9%)	3 (8.6%)	86 (56.6%)	
**Double expressor**					0.227
** Yes**	15 (42.9%)	2 (5.7%)	3 (8.6%)	72 (47.4%)	
** No**	9 (25.7%)	2 (5.7%)	4 (11.4%)	80 (52.6%)	
**COO**					0.013
** GCB**	11 (31.4%)	3 (8.6%)	5 (14.3%)	50 (32.9%)	
** Non-GCB**	12 (34.2%)	1 (2.9%)	2 (5.7%)	101 (66.5%)	
** NA**	1 (2.9%)	0 (0%)	0 (0%)	1 (0.6%)	

IPI, International Prognostic Index score; COO, cell of origin; GCB, germinal center B cell; HGBL-DH, double-hit high-grade B-cell lymphoma; HGBL-TH, triple-hit high-grade B-cell lymphoma; HGBL-NOS, high-grade B-cell lymphoma, not otherwise specified.

Compared with the non-HGBL group, the HGBL group was more likely to have a high-grade histopathological appearance, including necrosis, massive mitoses, or a “starry sky” appearance (p = 0.009). Other statistically significant differences between the HGBL and non-HGBL patients were bone marrow involvement (28.6% vs. 11.8%; p = 0.012) and extranodal involvement >1 (42.9% vs. 23%; p = 0.017). The SUVmax of HGBL patients was higher than that of non-HGBL patients (p = 0.045), which means that the functional metabolism of tumors in HGBL is far more active than in non-HGBL. The IHC analysis of MUM-1 expression level was negatively correlated with HGBL, with the expression level in the HGBL group significantly lower than that of the non-HGBL group (**Table 2**). As for the HGBL subcategory, bone marrow involvement was significantly associated with HGBL-DH/TH compared with non-HGBL-DH/TH. Also, the high-grade histomorphology appearance was significantly different between patients with HGBL and non-HGBL. In addition, we found that the protein expression level of c-MYC was superior with HGBL-DH/TH than that with non-HGBL-DH/TH by IHC analysis, but the MUM-1 protein expression level of the HGBL-DH/TH group was inferior to that of the non-HGBL-DH/TH group (**Table S4**).

**Table 2:** Comparison of clinical features between HGBL and non-HGBL.

Features	HGBL (n = 35)	Non-HGBL (n = 152)	p-Value
**High-grade histomorphology**			0.009
** Yes**	6	7	
** No**	29	145	
**WBC > ULN**			0.398
** Yes**	6	18	
** No**	29	134	
**β2 microglobulin > ULN**			0.771
** Yes**	14/28	51/96^*^	
** No**	14/28	45/96	
**Extranodal involvement**			0.057
** Yes**	28	96	
** No**	7	56	
**E > 1**			0.017
** Yes**	15	35	
** No**	20	117	
**BM involvement**			0.012
** Yes**	10	18	
** No**	25	134	
**Gastrointestinal involvement**			0.056
** Yes**	13	33	
** No**	22	119	
**CNS involvement**			0.876
** Yes**	4	16	
** No**	31	136	
**CD10**			0.070
** Positive**	14/34	39/152	
** Negative**	20/34	113/152	
**Ki67 ≥ 90%**			0.351
** Yes**	17/34	61/148	
** No**	17/34	87/148	
**EBER**			0.569
** Positive**	2/31	6/146	
** Negative**	29/31	140/146	
**Serum LDH**	650.63 ± 172.292	502.26 ± 48.685	0.587
**Bcl-2**	0.7265 ± 0.297	0.7733 ± 0.320	0.082
**Bcl-6**	0.9412 ± 0.239	0.9603 ± 0.196	0.622
**c-MYC**	0.4955 ± 0.043	0.4101 ± 0.224	0.052
**MUM-1**	0.6964 ± 0.416	0.8264 ± 0.341	0.019
**SUVmax**	16 ± 14.30	26 ± 20.73	0.045

WBC, white blood cell count; ULN, upper limit of normal; E, Extranodal involvemen; BM, bone marrow; CNS, central nervous system; EBER, the Epstein–Barr virus-encoded small nuclear RNA; LDH, serum lactate dehydrogenase; SUVmax, baseline whole-body maximum standardized uptake; HGBL, high-grade B-cell lymphoma.

^*^Some patients’ data were not available.

### Survival Prognosis

**Figure 1:**
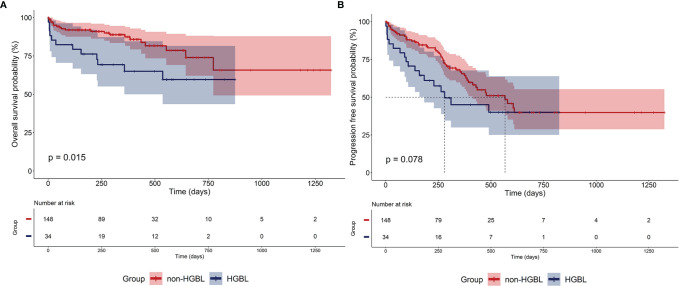
Outcomes of patients. **(A)** Overall survival (OS) and **(B)** progression-free survival (PFS) of high-grade B-cell lymphoma (HGBL) and non-HGBL patients.

The outcome of HGBL and non-HGBL patients undergoing induction chemotherapy is shown in **Figure 1**. Two patients harboring two types of tumors, one patient with HIV antibody positive, and two patients without survival data were excluded from the survival analysis. The Kaplan–Meier curves of OS revealed significant statistical differences between patients with HGBL and non-HGBL (p = 0.015). Compared with non-HGBL patients, HGBL patients had a more dismal prognosis and a trend toward superior median PFS. The median OS was not reached in all patients, while the median PFS in HGBL, non-HGBL, and all patients were 280, 567, and 490 days, respectively.

**Figure 2:**
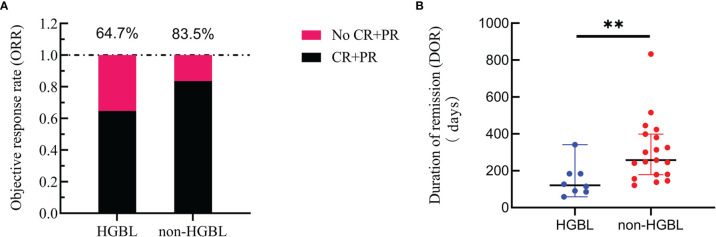
Curative effect. **(A)** Comparison of the objective response rate (ORR) between high-grade B-cell lymphoma (HGBL) patients and non-HGBL patients after induction chemotherapy with the R-CHOP regimen (p = 0.105). **(B)** Duration of remission (DOR) comparison of HGBL and non-HGBL patients from complete remission to relapse or death (**, p < 0.01).

Seventeen patients in the HGBL group and 79 in the non-HGBL group were given the R-CHOP regimen as the first-line treatment, of which 10 and 63 patients, respectively, achieved CR or PR after completing standard induction chemotherapy. The ORR of HGBL and non-HGBL patients treated with the R-CHOP regimen was 64.7% and 83.5%, respectively (p = 0.077, **Figure 2A**). Among 72 out of the 187 patients who achieved first CR, six cases with HGBL (including 4 cases of MYC-Bcl-6 HGBL-DH, one case of HGBL-TH, and one case of HGBL-NOS) and 17 cases with non-HGBL relapsed during maintenance treatment after the first remission. The DOR was significantly shorter in the HGBL group than in the non-HGBL group, with a median time of 121 vs. 258 days, respectively (p = 0.007; **Figure 2B**).

### Establishment of the Classification Model

**Figure 3:**
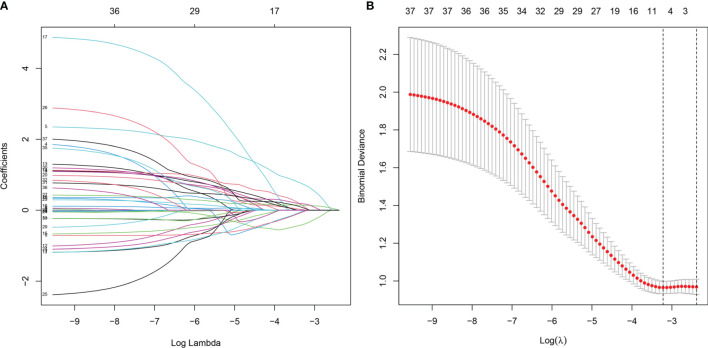
Clinical feature selection using the LASSO binary logistic regression model. **(A)** LASSO coefficient profiles of the 37 features. A coefficient profile plot was produced against the log(lambda) sequence. **(B)** Optimal parameter (lambda) selection in the LASSO model used eightfold cross-validation *via* minimum criteria. The partial likelihood deviance (binomial deviance) curve was plotted versus log(lambda). Dotted vertical lines were drawn at the optimal values by using the minimum criteria and the 1 SE of the minimum criteria (the 1 − SE criteria). LASSO, least absolute shrinkage and selection operator.

**Figure 4:**
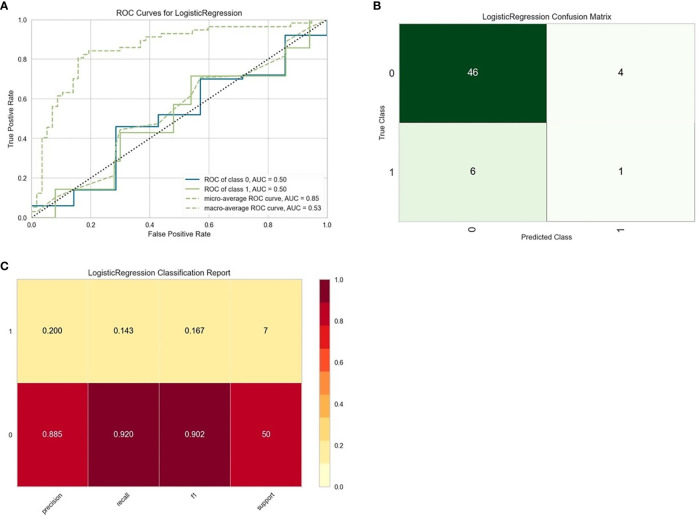
Evaluation of logistic regression models for predicting high-grade B-cell lymphoma (HGBL) in the test set. “Class 1” refers to the HGBL group, and “class 0” refers to the non-HGBL group. **(A)** The receiver operating characteristic (ROC) curves of the model. **(B)** The confusion matrix represents whether the classifier prediction is correct. **(C)** The precision, recall, F1 value, and support of the model.

Thirty-seven general clinical features collected were reduced to eight potential predictors with non-zero coefficients in the LASSO regression model (**Figure 3**). These potential predictors and other clinically significant factors in related studies were incorporated into the machine learning classification algorithm. After a comparison of the results of various models, a logistic binary regression model for predicting HGBL was established. The model showed high-grade histomorphology appearance (p = 0.012), advanced Ann Arbor stage (p = 0.007), LDH > upper limit of normal (ULN) (p = 0.045), and IPI risk group 3 or 4 (p = 0.003) as independent risk factors for HGBL (see **Table 3** for detailed equations). Evaluating the model’s effectiveness in the test set, the micro-average and macro-average AUC values of the ROC curve were 0.85 and 0.53, respectively. This model had high prediction efficiency for non-HGBL but was not excellent enough for HGBL (**Figure 4**).

**Table 3:** Logistic regression model for HGBL prediction.

Features	B	Wald	p	Exp (B)
**Gender**	0.608	0.772	0.380	1.836
**Age > 60**	0.012	0.133	0.715	1.012
**High-grade histomorphology**	2.693	6.302	0.012	14.774
**Ki67** ≥ **90%**	8.262	3.467	0.063	3874.165
**Bcl-2**	−2.279	3.650	0.056	0.102
**Bcl-6**	1.913	2.166	0.141	6.773
**c-MYC**	0.018	0.000	0.993	1.018
**Advanced Ann Arbor stage**	−1.375	7.161	0.007	0.253
**WBC > ULN**	0.064	0.599	0.439	1.067
**LDH > ULN**	−0.002	4.013	0.040	0.998
**β2 microglobulin**	0.158	0.984	0.321	1.171
**IPI score**	−1.456	2.646	0.104	0.233
**IPI risk group**	3.341	8.592	0.003	28.239
**Extranodal involvements**	0.398	3.738	0.053	1.489
**BM involvement**	1.710	2.673	0.102	5.531
**Constant**	−12.526	4.755	0.029	0.000

ULN, upper limit of normal; WBC, white blood cell count; LDH, serum lactate dehydrogenase; IPI, International Prognostic Index score; BM, bone marrow; HGBL, high-grade B-cell lymphoma.

**Figure 5:**
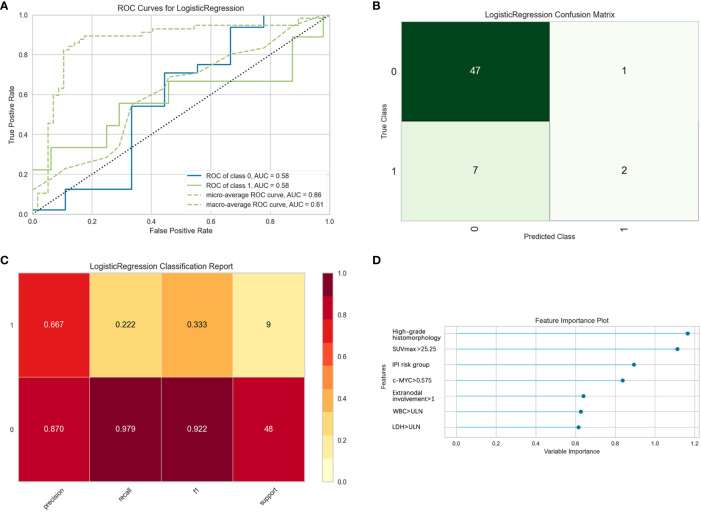
Evaluation of logistic regression models for predicting double-hit high-grade B-cell lymphoma (HGBL-DH) in the test set. “Class 1” refers to the HGBL-DH group, and “class 0” refers to the non-HGBL-DH group. **(A)** The receiver operating characteristic (ROC) curves of the model. **(B)** The confusion matrix represents whether the classifier prediction is correct. **(C)** The precision, recall, F1 value, and support of the model. **(D)** The bar plot represents the importance of clinical variables enrolled by the machine.

Likewise, a logistic binary regression model was established to predict HGBL-DH. Patients with high-grade histomorphology, SUVmax > 25.25, IPI risk group 3 or 4, c-MYC > 0.575, extranodal involvement >1, WBC > ULN, and LDH > ULN were more likely HGBL-DH. In the evaluation of the model’s effectiveness in the test set, the micro-average and macro-average AUC values of the ROC curve were 0.61 and 0.86, respectively (**Figure 5**). In view of the adverse impact of MYC rearrangement on prognosis, we also constructed a model to predict MYC rearrangement. After comparing with other models, we found that the Extreme Gradient Boosting classification model had the best AUC, precision, and recall rate, with the micro-average and macro-average AUC values being 0.81 and 0.70, respectively. In the feature importance plot, the variables according to the descending order in importance were high-grade histomorphology, c-MYC > 0.575, extranodal involvement >1, WBC > ULN, SUVmax > 25.25, IPI risk group 3 or 4, male, age > 60, LDH > ULN, and Bcl-6 overexpression positive (**Figure S2**).

**Figure 6:**
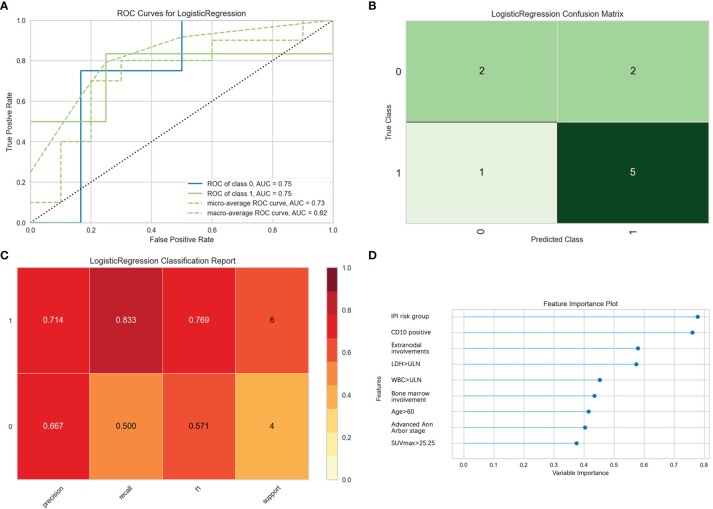
Evaluation of logistic regression model for predicting 1-year survival. “Class 1” refers to the death group, and “class 0” refers to the survival group. **(A)** The receiver operating characteristic (ROC) curves of the model. **(B)** The confusion matrix represents whether the classifier prediction in the test set is correct. **(C)** The precision, recall, F1 value, and support were used to evaluate the model’s prediction effectiveness. **(D)** The bar plot represents the importance of clinical variables enrolled by the machine.

Furthermore, the logistic binary regression model was chosen for the 1-year survival prediction. In the test set, the macro-average and micro-average AUC values of the ROC curve were 0.82 and 0.73, respectively. The validity of the model predicting death within 1 year was high, and the precision, the recall rate, and the F1 value of the test set were 0.714, 0.833, and 0.769, respectively. Patients with IPI risk group 3 or 4, CD10 positive, extranodal involvement, LDH > ULN, WBC > ULN, bone marrow involvement, age > 60, advanced Ann Arbor stage, and SUVmax > 25.25 had a higher risk of death within 1 year (**Figure 6**).

## Discussion

In the 2016 revision of the WHO classification of lymphoid neoplasms, HGBL is a newly introduced category of rare and heterogeneous invasive B-cell lymphoma that has recently received increasing attention clinically and in the literature. HGBL is comprised of two types, i.e., HGBL with MYC and Bcl-2 and/or Bcl-6 rearrangements (HGBL-DH or HGBL-TH) and HGBL-NOS, which replaced the category of B-cell lymphoma, unclassifiable, with features intermediate between DLBCL and BL (BCLU). Neither Ki-67 proliferation indices (23) nor double expressor is independently sensitive enough to distinguish the HGBL-DH or HGBL-TH cases. Some pathologists and clinicians have suggested that additional molecular genetic analysis should only be done in cases with the GCB phenotype or high-grade histomorphology features and those with MYC overexpression (4). Admittedly, using this protocol could save time and cost and may identify more HGBL patients. Nevertheless, it would inevitably miss some cases. For clinicians, accurate, widely available, and affordable methods are urgently needed.

Eleven of the 21 MYC/Bcl-6 HGBL-DH cases in our study were considered non-GCB phenotypes by the Hans classification. Furthermore, previous studies (24, 25) showed that MYC/Bcl-6 HGBL-DH was still observed in a large proportion of non-GCB phenotypes. Thus, it appears inappropriate to perform the FISH analysis only in cases with the GCB phenotype. At the same time, it is not advisable to perform a FISH test for all DLBCL cases. Hence, to explore which clinical characteristics can be regarded as diagnostic predictors and better understand this type of lymphomas, we analyze their clinical and pathologic features and establish a classification model.

Among the 24 HGBL-DH cases in our study, 21 patients had MYC and Bcl-6 rearrangements, and only 3 cases were MYC/Bcl-2 HGBL-DH. Interestingly, we found that MYC/Bcl-2 HGBL-DH constitutes the majority of HGBL-DH in most European and American studies (16, 26–29), such as in the United States, France, and Canada. In contrast, some studies from China’s southern regions like the Guangdong and Taiwan Provinces (30, 31) showed that MYC/Bcl-6 HGBL-DH is the most common. We consider that it may be due to geographical differences or the relatively small number of specimens. All 3 cases with MYC/Bcl-2 HGBL-DH had the GCB phenotype, which shows similarity to HGBL-TH in terms of COO and immunophenotype, in agreement with those earlier studies in the literature (32, 33). In our study, almost every HGBL-TH case had CD10 overexpression, while it also often was observed in MYC/Bcl-2 HGBL-DH, which was consistent with those reported results (33, 34).

For HGBL, the standard treatment was not yet established, and R-CHOP remains the basis of therapy. Some reports (35, 36) showed that higher-intensity chemotherapy such as R-DA-EPOCH can prolong PFS and OS than R-CHOP in HGBL-DH patients. Still, the retrospective analysis of Landsburg et al. (27) showed no differences in OS between R-DA-EPOCH and R-CHOP. Moreover, due to insufficient data on HGBL-TH and HGBL-NOS patients, it is still necessary to develop new treatments to improve the prognosis of these patients. In our study, the HGBL group also showed a dismal prognosis, and the classical R-CHOP regimen has also been shown to have lower efficacy. In addition, patients treated with R-CHOP combined with lenalidomide, ibrutinib, or chidamide (R-CHOP + X) regimens exhibited initial curative effects. However, this approach will need to be tested in more patients.

Classification models were established to predict the diagnosis and prognosis of HGBL by machine learning algorithms in this study. The Extreme Gradient Boosting approach had the highest AUC, while the random forest classification model had the highest accuracy. However, in case of the good interpretability of the logistic regression model, whose AUC and precision are also reliable, we chose to build the logistic binary regression model to predict HGBL. Some models showed high effective predictive ability, including HGBL-DH and MYC rearrangement. They indicated that we should focus on SUVmax, IPI risk group, c-MYC overexpression, Bcl-6 overexpression, extranodal involvement, WBC, and LDH as references when considering whether FISH screening was recommended. It is noteworthy that the 1-year survival prediction model had a high AUC, precision, and recall rate. This model could be a helpful prognosis evaluation method for clinicians. However, because of the complexity of diagnosis of HGBL and the limited number of cases in a single research center, the bias of these models was hard to certify. Multicenter studies need to be carried out in the future to improve the accuracy of the models further.

In summary, HGBL is a new category of highly aggressive B-cell malignancies characterized by laborious diagnosis and poor effects of therapy. Our study identified several independent risk factors for the diagnosis of HGBL. Prediction models contribute to clinicians making a comprehensive diagnosis and evaluating the prognosis more accurately. Otherwise, R-CHOP, as the most frequently used first-line treatment, was considered to have dismal efficacy for HGBL in our study. A standard induction therapeutic regimen is urgently needed to ameliorate the poor outcome.

## Data Availability Statement

The original contributions presented in the study are included in the article/**Supplementary Material**. Further inquiries can be directed to the corresponding authors.

## Ethics Statement

The studies involving human participants were reviewed and approved by Fujian Medical University Union Hospital Ethics Committee. The patients/participants provided their written informed consent to participate in this study.

## Author Contributions

TY and JH designed and performed the study. HK, HZ, and XZ collected the data. HK, JR, and XL collected the pathological specimens. MJ and LC performed the IHC and FISH analyses. HK, HZ, and LL performed the statistical analyses. HZ, JR, and TY interpreted the results and developed the initial manuscript draft. JZ, ZZ, XZ, and ZC contributed to patient management. All authors contributed to manuscript revisions and approved the final version for publication. TY and JH had full access to all the data and had final responsibility for the decision to submit it for publication.

## Funding

This work was funded by the National Natural Science Foundation of China (81870138, U2005204), Startup Fund for Scientific Research Project of Fujian Medical University (2020QH2021), National Key Clinical Specialty Discipline Construction Program (2021-76), and Clinical Research Center for Hematological Malignancies of Fujian Province (2020Y2006).

## Conflict of Interest

The authors declare that the research was conducted in the absence of any commercial or financial relationships that could be construed as a potential conflict of interest.

## Publisher’s Note

All claims expressed in this article are solely those of the authors and do not necessarily represent those of their affiliated organizations, or those of the publisher, the editors and the reviewers. Any product that may be evaluated in this article, or claim that may be made by its manufacturer, is not guaranteed or endorsed by the publisher.
