# Supplementary material for: Machine Learning Models for the Diagnosis and Prognosis Prediction of High-Grade B-Cell Lymphoma
Source: Front Immunol. 2022 May 24;13:919012. doi: 10.3389/fimmu.2022.919012 (PMC9171399; doi:10.3389/fimmu.2022.919012)
Supplement: Supplementary file 1 [file DataSheet_1.pdf]

Supplementary Material

Supplementary material and methods

The indicators of prediction models evaluating

For the confusion matrix, the prediction label of the model and the real label can be divided into four parts according to the distribution model of confusion matrix:

TP (True Positives), the model predicted 1 and the real sample was also 1;
TN (True Negatives), the model prediction and True sample both were 0;
FP (False Positives), the model predicted 1 and the real sample was 0;
FN (False Negatives), the model predicted 0 and the real sample was 1.

And Precision is the ratio of TP and TP+FP. Recall, namely sensitivity, is the ratio of TP and TP+FN. F1 value represents the harmonic mean between precision and recall rate. Support is the number of classified samples or the total number of samples in the testing set.

Supplementary table and figure

Table S1： Ann-Arbor staging revised by Cotswolds *et al.* in 1989

Stage	Definition
**I**	Single lymph node area (I) or focal single extranodal organ (IE) invasion
**II**	Invasion of lymph nodes (II) or focal single extranodal organ and regional lymph nodes on the same side of the diaphragm with or without other lymph nodes on the same side of the diaphragm (IIE)
**III**	Simultaneous invasion of diaphragmatic lymph nodes (III) with focal extranodal organs (IIIE), spleen (IIIS) or both (IIIE + S)
**IV**	Diffuse (multifocal) single or multiple extranodal organs were invaded, with or without associated lymph node enlargement, or isolated extranodal organs were invaded, with distant (non-regional) lymph node enlargement. If liver or bone marrow is involved, even limitations are stage IV

Note: Spleen and Wechsler ring lymph tissues were marked as non-extranodal areas. Extranodal lesions were classified as stage IV, including bone marrow, lung, bone, or liver involvement.

Table S2： International prognostic index (IPI) for lymphoma and age adjusted IPI (aaIPI)

Factor	0 point	1 point
**Age**	⩽ 60	＞ 60
**ECOG physical fitness score**	0-1	⩾ 2
**Ann Arbor staging**	I-II	III-IV
**Extranodal site of invasion**	＜ 2	⩾ 2
**Increased LDH**	No	Yes

Note: For patients under 60 years old, the age adjusted IPI score was calculated with the lesion as stage III or IV, the upper limit of LDH > normal value, and the ECOG score ⩾ 2 as the evaluation standard. The IPI and aaIPI score was converted into risk group. The details as follows:

**Risk group**	**IPI score**	**aaIPI score**
	**Age ＞ 60 patients**	**Age ⩽ 60 patients**
**Low (1)**	0 or 1	0
**Low intermediate (2)**	2	1
**High intermediate (3)**	3	2
**High (4)**	4 or 5	3

Table S3. Abnormal karyotypes for cases with available cytogenetic data

**HGBL (n=18)**	**Abnormal karyotype**	**ACA score**
**Case#19**	45, X, -Y [1]/52-59, idem, +X, +1, der(1)?t(1;9)(p13;p11), +2, +5, +7, +8, +9, +11, +12, +14, add(14)(q32). +17, +mar1, +mar2[CP19]	16
**Case#31**	46, XY ins (1;?) (q11;?) [19]/46, XY [1]	1
**Non-HGBL (n=69)**	**Abnormal karyotype**	**ACA score**
**Case#47**	47-48, XY, del (6) (q13q23), add (7) (p11), -19, +mar1, +mar2, +mar3 [CP4] /46, XY, [26]	6
**Case#63**	44-49, XX,del(1)(q24q41),+3,dup(3)(p21),+6,del(6)(q16q21)×2,+7,add(11)(q25), add(14)(q24),del(19)(q13),der(20)t(1;20)(q11;q13),+mar1,+mar2,+mar3[CP12] /46, XX[4]	13
**Case#101**	47, X, -X, t(1;16) (p10,q10), del(1)(p12), +der(1)del(1)(p13), +der(3)inv(3) (q12q27), -10, +der(11)add(11)(q23), del(13)(q21), del(15)(q23), +18, -19[5] /46, XX [13]	11
**Case#107**	49-52, XY, +X, t(9;12) (p22;p12),+10,+12,+13,-14, +15, t(17;17) (q25;q22), +18, +r[CP9]/46, XY[11]	9
**Case#159**	47, XY, +Y [3]/46, XY [17]	1
**Case#165**	48-49, XX, +3, add (14) (q32), inc [CP2]/46, XY [12]	3
**Case#174**	46, XY, t(3;3)(p26;q22),del(6)(q15),der(6)[1]/46,XY[19]	3

ACA, additional cytogenetic abnormalities; CP, composite karyotype.

Table S4. Comparison of clinical features between HGBL-DH/TH and non-HGBL-DH/TH

Features	HGBL-DH/TH (n=28)	Non-HGBL-DH/TH (n=159)	P value
**High-grade histomorphology**			0.001
**Yes**	6	7	
**No**	22	152	
**BM involvement**			0.029
**Yes**	8	20	
**No**	20	139	
**MUM-1**	0.535±0.464	0.758±0.410	0.012
**c-MYC**	0.5404±0.232	0.4106±0.229	0.008

Abbreviation: BM: bone marrow.

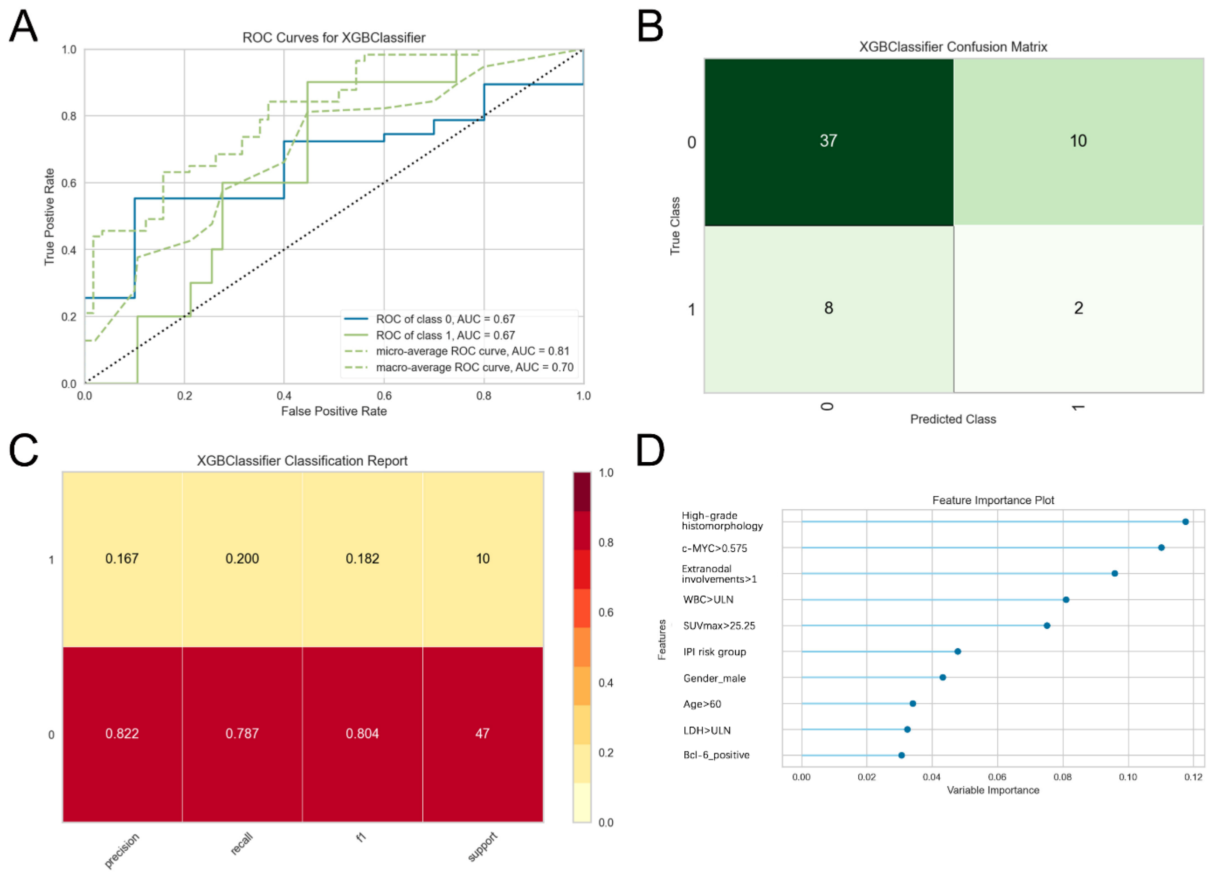

Figure S1. Evaluation of Extreme Gradient Boosting models for predicting MYC rearrangement in the test set. "class 1" refers to the MYC rearrangement group and "class 0" refers to the non-MYC rearrangement group. (A) The ROC curves of the model. (B) The confusion matrix represents whether the classifier prediction is correct. (C) The precision, recall, F1 value and support of the model. (D) The bar plot represents the importance of clinical variables enrolled by the machine.
